# Leveraging real-time genomic surveillance to combat infectious diseases and antimicrobial resistance in cancer patients

**DOI:** 10.3389/fpubh.2026.1763512

**Published:** 2026-02-13

**Authors:** Yuemei Hong, Despoina Kalfakakou, Io Hong Cheong, Zisis Kozlakidis

**Affiliations:** 1 International Agency for Research on Cancer, World Health Organization, Lyon, France; 2 Department of Medicine, Division of Precision Medicine, NYU Grossman School of Medicine, New York, NY, United States; 3 Hainan International Medical Center, Shanghai Jiao Tong University School of Medicine, Shanghai, China; 4 State Key Laboratory of Oncogenes and Related Genes, Center for Single-Cell Omics, School of Public Health, Shanghai Jiao Tong University School of Medicine, Shanghai, China

**Keywords:** antimicrobial resistance, cancer, healthcare-associated infections, infectious diseases, real-time genomic surveillance

This opinion article outlines four essential pillars for integrating real-time genomic surveillance into cancer care: the need for Laboratory infrastructure, Bioinformatics and Data Flow/Integration, as well as the necessary considerations for costs. These elements can enable oncology units to transition toward a more proactive model of infection and AMR monitoring that informs antimicrobial stewardship, strengthens outbreak preparedness, and enhances patient safety.

## Introduction

Infections and antimicrobial resistance (AMR) represent major threats to cancer patients, with global analyses showing a high burden of multidrug-resistant (MDR) pathogens in this population (1). Cancer patients exhibit a significantly elevated susceptibility to drug-resistant infections, with incidence rates reaching up to three times those observed in the non-cancer population (2). Moreover, the repeated use of broad-spectrum antibiotics during a patient's cancer treatment(s) can create strong selection pressures that drive the emergence and expansion of multidrug-resistant pathogens, thereby narrowing therapeutic options and posing major clinical challenges (3, 4). Large-scale cohort studies and systematic reviews have demonstrated that bacterial infections and AMR are now major drivers of morbidity in cancer care. Among solid tumor patients with bloodstream infections, MDR Gram-negative bacteria account for the majority of episodes and are consistently associated with increased intensive-care admission, treatment failure, and short-term mortality (5–8). In patients with febrile neutropenia, particularly those with hematological malignancies, bloodstream infections caused by MDR bacteria such as *Escherichia coli* and *Klebsiella pneumoniae* are associated with markedly elevated case-fatality (9, 10). The substantial prevalence of *Enterococcus faecium, Staphylococcus aureus, Klebsiella pneumoniae, Acinetobacter baumannii, Pseudomonas aeruginosa, Enterobacter spp*. (ESKAPE) pathogens in oncology populations underscores the urgent need to address infection and antimicrobial resistance (11–16).

Thus, tackling AMR will require a high sensitivity identification and sustained surveillance of infectious diseases within the cancer population. However, global AMR surveillance remains critically undermined by incomplete coverage and fragmented data streams, particularly in large parts of Africa, Southeast Asia, and Latin America where systematic, population-level monitoring is often absent, forcing reliance on modeled estimates with a reported wide uncertainty (17–19). Moreover, surveillance continues to rely predominantly on slow, low-resolution conventional diagnostics such as blood cultures (standard incubation is 5 days) which have lower sensitivity than molecular methods (20). The limitations of these methods can thus fail to deliver information fast enough to inform immediate clinical decisions or support antimicrobial stewardship and can often lack the resolution required for precise pathogen tracking (21).

Yet, the molecular methods need predefined resistance targets and cannot fully resolve transmission events without related clinical and epidemiological information (22). The extensive experiences from the real-time genomic surveillance over the last decade, by utilizing whole genome sequencing (WGS) and metagenomics, represent available solutions to the growing threats of infectious diseases and AMR-tracking by revealing transmission networks, tracing high-risk lineages, and enabling early detection of emerging resistance (23, 24). Next-generation sequencing technologies accelerate our ability to detect and characterize antimicrobial resistance both at a population (25) as well as at an individual level within clinical settings (26–28).

Despite this potential, even highlighted for certain infectious diseases by the World Health Organization (WHO), such as for tuberculosis (29), the global adoption of NGS remains fragmented and inconsistent, and is rarely systematically integrated into national monitoring programs, particularly in resource-limited settings due to unsustainable funding, insufficient laboratory capacity, and a lack of quality assurance (30). This failure to operationalize comprehensive genomic surveillance perpetuates significant data deficits regarding AMR prevalence in high-risk populations. Consequently, morbidity remains systematically underestimated, and critical windows for early clinical intervention are MDR, exacerbating the public health impact of resistant infections. This is especially pronounced in cancer patient populations, where AMR surveillance is equally non-systematic and fragmented. Considering potential avenues to ameliorate the current situation, this opinion piece proposes four key pillars that can leverage real-time genomic surveillance for effectively monitoring AMR emergence in cancer patients.

## Leveraging metagenomics and NGS for targeted surveillance

To leverage metagenomics and NGS, a framework is essential for aligning different NGS sequencing approaches with clinical risk, sample type, and diagnostic complexity. Additionally, such a framework would need to consider the context-dependent balance between diagnostic resolution and cost-effectiveness.

### Low(er)-cost active surveillance

The utilization of low-depth shotgun metagenomics provides a highly cost-efficient mechanism for the high-frequency, longitudinal monitoring of patient colonization dynamics (31, 32). Unlike resource-intensive deep sequencing, this scalable approach allows for affordable and replicable profiling, e.g., of the gut microbiome throughout treatment of cancer patients (33). However, the risk that low-depth metagenomics may limit the ability to perform transmission analyses should be taken into account. Thus, a balance is required at each context, between the need to lower costs and the introducing a tradeoff in which AMR detection may not outperform existing diagnostic or surveillance methods. The current experiences with shallow shotgun sequencing (in clinical and mock microbiome samples) demonstrate that a depth of 0.5 million sequences per sample was sufficient to produce a quality similar level of species and functional profiles to that of deep WGS sequencing such as understanding which species and functions are present and identifying biomarkers related to outcomes (31, 32). This surveillance is sufficiently sensitive to capture the subtle expansion of AMR genes. By identifying the proliferation of resistant clones or specific mutations prior to clinical manifestation, or at specific timepoints of the patient journey, such as near the time of diagnosis, this strategy allows clinicians to adjust prophylactic regimens or initiate preemptive stewardship measures before life-threatening infections occur (34). Costs can be reduced further by a shift to more conventional sequencing approaches when NGS is not required. Additionally, in the case of pathogens with high viral load samples where sensitivity rates are low, sample pooling strategies with validated deconvolution can also enhance efficiency.

Effective routine surveillance also requires the establishment of a local genomic database by clinical microbiology laboratories, complemented by environmental samples from cancer patient wards. Such a database enables high-resolution phylogenetic analysis, which is essential for distinguishing persistent institutional clones from imported strains (35). Currently, environmental sampling, e.g. for high-touch surfaces, is typically recommended only in specific outbreak contexts, where results directly inform targeted interventions. However, shallow-depth shotgun metagenomics can also be leveraged in highly targeted environmental sampling guided interventions, in the latter case able to unveil ecological niches of microbes and antibiotic resistance genes (36). Consequently, such an approach can guide evidence-based outbreak responses, allowing infection control teams to implement targeted containment strategies such as specific environmental decontamination, or staff screening (37).

The maximization of pathogen genomics utility is contingent upon the existence of a functional laboratory infrastructure and coordinated surveillance networks. In settings with severe resource limitations, the immediate adoption of genomic technologies is often unfeasible. For existing laboratory infrastructures, experts from the WHO and other institutions recently released a tool to assist with commodity forecasting and costing for pathogen genomics. Such a tool can be used for national budgeting purposes (38). As hospitals operate within fixed budgets and often see no direct financial or operational return on such investments, the cost of WGS/NGS can be embedded into national health budgets as part of broader policy-driven disease control and preparedness programs. However, the affordable cost threshold depends on context and the ability of countries' Ministries of Health to advocate for domestic funding and enhanced routine surveillance. A list of priority pathogens has been developed by WHO to enhance outbreak preparedness by advocating for investments in research, development, and innovation in medical countermeasures. To this end, the prioritized pathogens included under routine NGS/WGS surveillance to yield measurable benefit for cancer patients could be a subset from this priority list, providing a global comparable reference point.

### A unified laboratory framework

The ability to shift from lower to increased diagnostic resolution in a stepwise fashion and within a clinical environment, predisposes that all of these options would be running in parallel. Thus, the flexibility provided by a tiered diagnostic testing can be more readily achieved in larger, tertiary clinical units, where the available infrastructure, consumables and expertise are most likely to be present (39). In terms of cancer care, several cancers are treatable only within such units, and therefore the additional tiered approach would be complementary to existing laboratory functions. An example of such a tiered approach would be to first use low-cost rapid tests (e.g., antigen tests, CRP/PCT for triage), followed by targeted PCR for likely pathogens, and finally NGS sequencing for complex or unresolved cases. The NGS data can be compared to the sequenced environmental samples derived from the same hospital to provide a more comprehensive interpretation of results. Comparing NGS data from clinical samples with environmental surveillance data enables differentiation between endogenous infection and exogenous acquisition and confirmation of indirect transmission pathways. This has been shown in the case of identifying sources of environmental contamination (40), as well as in the reconstruction of the potential outbreak transmission route of drug-resistant pathogens in a hospital environment (37) and within acceptable operational costs (41).

However, to achieve data interoperability, a centralized, standardized genomic analysis pipeline should be implemented across all participating sampling locations within the same hospital. This necessitates either the use of existing, or the establishment of harmonized standard operating procedures (SOPs) covering the entire workflow from DNA extraction, library preparation to minimize technical batch effects and experimental bias (42). Crucially, on the computational side, the network should also adopt containerized bioinformatic workflows to support the use of identical software versions and parameter settings across the same functions of different hospital departments (43). In parallel, adopting FAIR (Findable, Accessible, Interoperable, Reusable) data principles would further strengthen interoperability across departments by ensuring that genomic datasets are consistently organized, documented, and easily integrated into shared analytical frameworks (44). By aligning sampling, laboratory and bioinformatics standards, such an approach will guarantee rigorous quality control. This unified laboratory framework ensures that resistance profiles and transmission clusters identified in one patient and/or ward are directly comparable to those in another, facilitating accurate local AMR surveillance.

### Flexible bioinformatics capacities

The genomic data streams from cancer patients should be consolidated into a centralized, secure data platform that integrates sequencing outputs with clinical and epidemiological metadata, such as antibiotic exposure history, immune status, and treatment outcomes (45). This integration can power the deployment of near real-time decision-support dashboards, which visualize transmission networks and, when sufficient data is available, leverage machine-learning methods to generate automated early-warning alerts for high-risk clones or anomalous resistance profiles (46), ultimately transforming raw genomic data into actionable intelligence that guides infection control interventions and therapeutic adjustments. However, such flexible bioinformatics infrastructures capable of integrating genomic data with electronic clinical records are difficult to implement in general, and even more so in middle-income settings. Successful models of twinning between high-income and resource-restricted facilities, have shown that technology transfer, local adaptation and implementation of surveillance and bioinformatics capacities are feasible (47). While these models are indeed successful, they are slow and limited by funding support, as well as the numbers and availabilities of willing institutions and experts.

### Data flow and integration, while upholding data privacy and biosecurity

Data privacy and biosecurity are increasingly rigorously regulated globally, which means that any suggested data flow and integration need to be aligned with existing regulatory requirements (48, 49). While such structures exist for infectious disease control units, it may need to be extended for cancer units too—or for the latter to update their data handling to include a clear mention for the use of infectious disease data from cancer patients. Access to this high-resolution data is typically governed by institutional review boards, granting full access to authorized clinical and public health personnel while providing anonymized, aggregated data for broader research (50). However, in the case of cancer patients, a dedicated interdisciplinary oversight would be required, bridging the specialties of oncology, epidemiology and infectious diseases—leveraging the experiences in which antimicrobial stewardship has been integrated with surveillance in several clinical settings (51, 52). This consideration for continuous data flow and integration is warranted both as cancer patients represent a susceptible population, as well as often serve as long-term reservoirs for multidrug resistant organisms due to prolonged hospitalization and heavy antibiotic selection pressure (53).

## Discussion

The technological development of NGS, coupled by the considerable savings that can be achieved by high-throughput sequencing, has enabled methods that were designed primarily for infectious diseases surveillance at a population level, to be applied within clinical settings. These approaches can also be extended to patient groups, such as cancer patients, where infectious diseases are taking an increasing toll on patient survival. By leveraging metagenomics for the longitudinal monitoring of patient colonization dynamics and applying NGS for the high-resolution tracking of nosocomial clones, this combination can establish a complementary framework that overcomes the sensitivity limitations of traditional culture. An integrated genomic surveillance network, complemented by locally sourced environmental samples can bridge the gap between laboratory data and clinical practice. However, the sustainable operationalization of network hinges on four key elements (Laboratory infrastructure, Cost, Bioinformatics and Data Flow/Integration), as identified originally for NGS implementation in general (23), and being equally as applicable to cancer patient settings. Moreover, specialized technical capability is needed for implementing harmonized laboratory standards under a unified framework and for flexible bioinformatic pipelines. However, leveraging such a comprehensive surveillance comes with considerable challenges for additional infrastructure, staff, funding and training capacities.

Genomic surveillance can only reduce transmission if healthcare settings are able to act on the information generated. This requires a trained and sufficiently resourced workforce capable of interpreting transmission clusters and implementing targeted interventions. Currently, infection preventionists generally receive little formal training in how to operationalize genomic data, and the increased detection of transmission events may substantially increase workload. Thus, the current deficit in genomic literacy among infection control teams, particularly within high-stakes oncology settings needs to be addressed. For example, this can include the development of targeted, short-format training modules designed to help infection control teams to interpret phylogenetic cluster reports and prioritize interventions accordingly (54, 55). Another example can be the integration of algorithms to filter noise and prioritize clusters based on risk factors, ensuring that alerts are triggered only by events requiring immediate attention (56, 57) reducing a potential increase in workload to the absolutely necessary. Moreover, these requirements can be partly ameliorated by the participation of cancer centers from tertiary hospitals as reporting centers for regional shared surveillance networks. This means that hospital units in the same region may have access to additional sequencing capacity, data platforms and/or epidemiological expertise, avoiding duplication where possible and lowering per-site surveillance costs.

## Conclusion

The real-time genomic surveillance represents a transformative opportunity to reshape infectious disease and antimicrobial resistance management in cancer care. By leveraging such a technology, oncology units can move beyond the inherent limitations of culture-based diagnostics toward a more proactive, high-resolution understanding of pathogen dynamics. However, the effectiveness of genomic surveillance extends far beyond sequencing technologies alone. Its clinical value depends on harmonized laboratory standards under a unified laboratory framework, on interoperable bioinformatic pipelines, and an integrated data ecosystem that links genomic findings with epidemiological and patient metadata. These should be supported by appropriately trained staff. Decision-support dashboards and at a later point machine learning tools can transform these rich data streams into real-time intelligence, supporting antimicrobial stewardship and informing precision infection control strategies. Implementing such a system within routine cancer care requires a robust regulatory and ethical framework that ensures privacy and defines transparent governance structures for data use and oversight. This opinion manuscript provides an oversight into necessary actions with a specific perspective for cancer patients.
